# A novel monoclinic phase of impurity-doped CaGa_2_S_4_ as a phosphor with high emission intensity

**DOI:** 10.1107/S1600536812019113

**Published:** 2012-05-12

**Authors:** Akihiro Suzuki, Takeo Takizawa, Chiharu Hidaka, Nomura Shigetaka, Ittetsu Kitajima

**Affiliations:** a Department of Physics,College of Humanities and Sciences, Nihon University, 3-25-40 Sakura-josui, Setagaya-ku, Tokyo 156-8550, Japan; b Department of Electrical Engineering, Faculty of Engineering, Tokyo University of Science, 1-14-6 Kudan-kita, Chiyoda-ku, Tokyo 102-0073, Japan

## Abstract

In the solid-state synthesis of impurity-doped CaGa_2_S_4_, calcium tetra­thio­digallate(III), a novel phosphor material (denominated as the *X*-phase), with monoclinic symmetry in the space group *P*2_1_/*a*, has been discovered. Its emission intensity is higher than that of the known ortho­rhom­bic polymorph of CaGa_2_S_4_ crystallizing in the space group *Fddd*. The asymmetric unit of the monoclinic phase consists of two Ca, four Ga and eight S sites. Each of the Ca and Ga atoms is surrounded by seven and four sulfide ions, respectively, thereby sharing each of the sulfur sites with the nearest neighbours. In contrast, the corresponding sites in the ortho­rhom­bic phase are surrounded by eight and four S atoms, respectively. The photoluminescence peaks from Mn^2+^ and Ce^3+^ in the doped *X*-phase, both of which are supposed to replace Ca^2+^ ions, have been observed to shift towards the high energy side in comparison with those in the ortho­rhom­bic phase. This suggests that the crystal field around the Mn^2+^ and Ce^3+^ ions in the *X*-phase is weaker than that in the ortho­rhom­bic phase.

## Related literature

For the crystal structure of the ortho­rhom­bic form of CaGa_2_S_4_, see: Eisenmann *et al.* (1983 ▶). For the emission properties of CaGa_2_S_4_, see: Peters & Baglio (1972 ▶); Georgobiani *et al.* (1995 ▶); Bessière *et al.* (2004 ▶); Takizawa & Hidaka (2008 ▶). For synthetic details, see: Boitier *et al.* (2009 ▶); Obonai *et al.* (2009 ▶). For the Mn^2+^ emission properties in various compounds, see: Barthou *et al.* (1994 ▶); Provenzano & White (1991 ▶); Wang *et al.* (2003 ▶). For crystallographic background, see: Carruthers & Watkin (1979 ▶).

## Experimental

### 

#### Crystal data

CaGa_2_S_4_
*M* *_r_* = 307.76
Monoclinic, 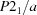

*a* = 6.841 (3) Å
*b* = 27.069 (9) Å
*c* = 7.231 (3) Å
β = 109.413 (5)°
*V* = 1262.9 (8) Å^3^
*Z* = 8
Mo *K*α radiation
μ = 10.52 mm^−1^
*T* = 270 K
0.10 × 0.10 × 0.05 mm

#### Data collection

Rigaku Saturn724+ diffractometer
Absorption correction: multi-scan (*REQABS*; Jacobson, 1998 ▶) *T*_min_ = 0.417, *T*_max_ = 0.591
8869 measured reflections
2869 independent
256 reflections with *F* ^2^ > 2σ(*F* ^2^)
*R*_int_ = 0.047

#### Refinement

*R*[*F* ^2^ > 2σ(*F* ^2^)] = 0.038
*wR*(*F* ^2^) = 0.050
*S* = 1.21
2869 reflections
127 parameters
Δρ_max_ = 2.64 e Å^−3^
Δρ_min_ = −3.10 e Å^−3^

### 

Data collection: *CrystalClear* (Rigaku/MSC and Rigaku, 2006 ▶); cell refinement: *CrystalClear*; data reduction: *CrystalStructure* (Rigaku/MSC and Rigaku, 2006 ▶); program(s) used to solve structure: *SIR92* (Altomare *et al.*, 1994 ▶); program(s) used to refine structure: *CRYSTALS* (Betteridge *et al.*, 2003 ▶); molecular graphics: *VESTA 3* (Momma & Izumi, 2011 ▶); software used to prepare material for publication: *CrystalStructure* (Rigaku/MSC and Rigaku, 2006 ▶).

## Supplementary Material

Crystal structure: contains datablock(s) global, I. DOI: 10.1107/S1600536812019113/wm2605sup1.cif

Structure factors: contains datablock(s) I. DOI: 10.1107/S1600536812019113/wm2605Isup2.hkl

Additional supplementary materials:  crystallographic information; 3D view; checkCIF report

## Figures and Tables

**Fig. 1.**
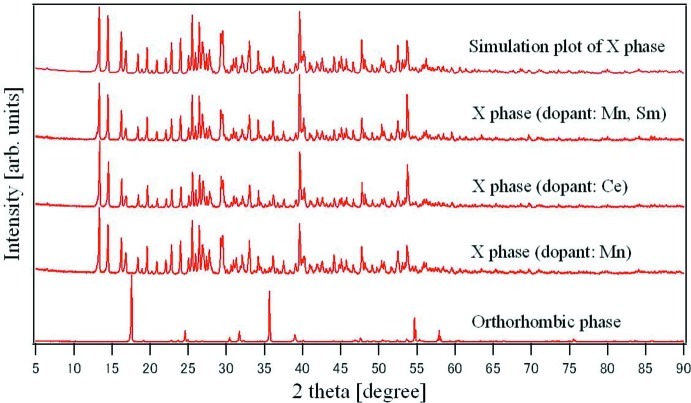
Experimental and siumlated X-ray powder diffraction patterns of the X-phase of CaGa2S4 together with the known orthorhombic form for comparison.

**Fig. 2.**
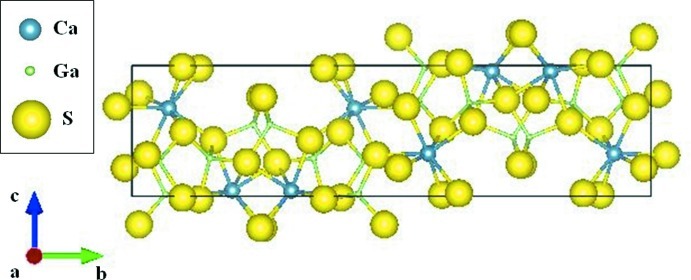
The unit cell of the X-phase of CaGa2S4 viewed along [100].

**Fig. 3.**
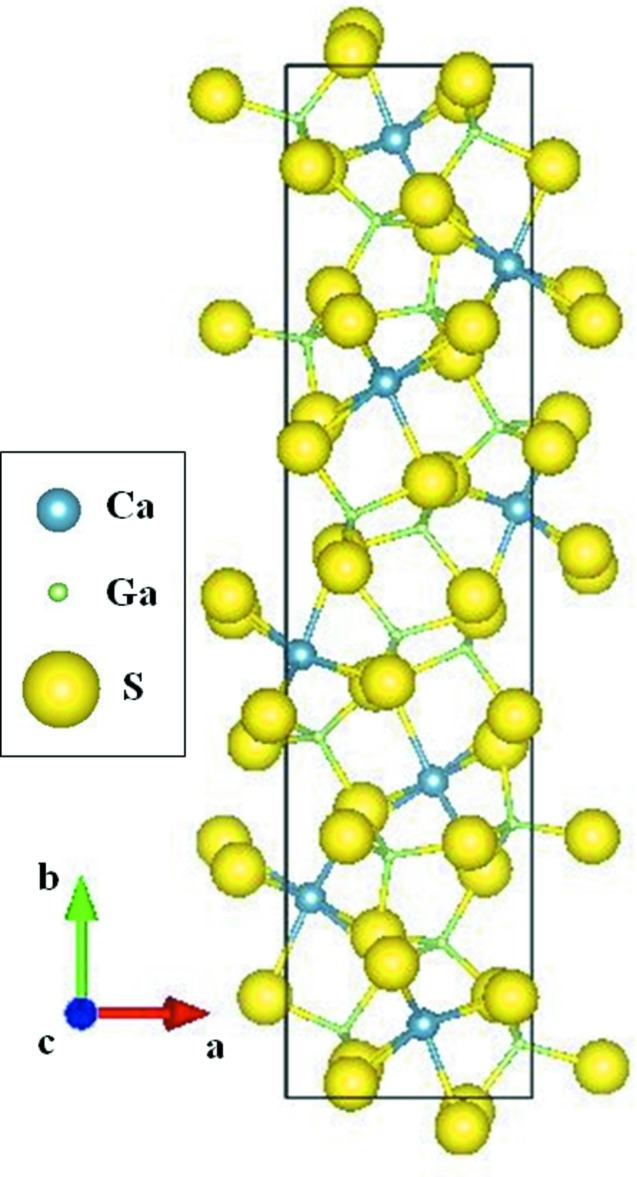
The unit cell of the X-phase of CaGa2S4 viewed along [001].

**Fig. 4.**
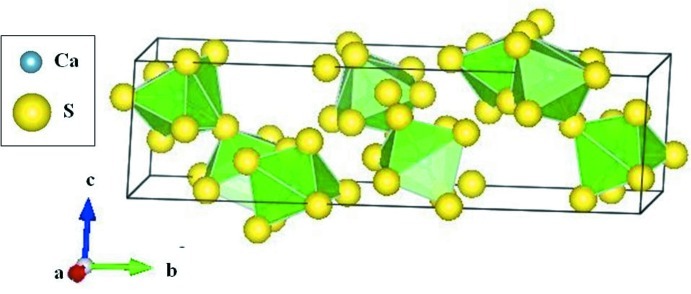
The environment of the cation sites in the X-phase: Ca sites.

**Fig. 5.**
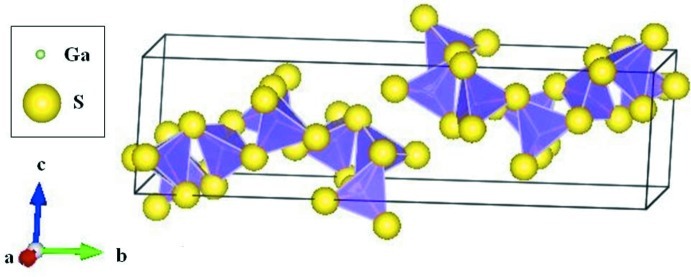
The environment of the cation sites in the X-phase: Ga sites.

## supplementary crystallographic information

### Comment

The alkaline earth thiodigallates *M*Ga_2_S_4_ (*M* = Ca, Sr, Ba) have been studied as candidates for phosphor host materials because of their various emissions when doped with a rare earth element (Peters & Baglio, 1972; Georgobiani *et al.*, 1995; Bessière *et al.*, 2004; Takizawa & Hidaka, 2008). Especially, CaGa_2_S_4_ doped with Ce^3+^ and Eu^2+^ shows strong blue and green emissions. This compound normally belongs to the orthorhombic system, crystallizing in space group *Fddd* (Eisenmann *et al.*, 1983). However, in the synthesis of CaGa_2_S_4_ using solid state reactions, a novel phosphor (we call it the *X*-phase) has unexpectedly appeared. In this paper, the crystal structure of the *X*-phase investigated by powder and single-crystal X-ray diffraction (XRD) measurements is reported.

Powder XRD patterns of the *X*-phase are shown in Fig. 1. This phase happened to be generated when doped with one of the impurities such as Mn, Ge, Sn, La, Ce, Nd, Sm, Dy, intentionally incorporated as an activator of fluorescence or phosphorescence. Thus it is expected that the *X*-phase is strongly related with the dopant. However, detailed conditions how to prepare the *X*-phase from a directed preparation route have not been clarified yet. From the analysis of the single-crystal XRD data, the chemical formula of the compound in the *X*-phase was shown to be CaGa_2_S_4_. The simulation of the powder XRD pattern corresponds well to the experimental pattern as shown in Fig. 1. Hence we can conclude that the *X*-phase is a newly found polymorph of CaGa_2_S_4_.

The unit cell of the *X*-phase is shown in Figs. 2 and 3; the asymmetric unit consists of two Ca sites, four Ga sites and seven S sites. Figs. 4 and 5 show the environment of the cation sites in the unit cell. In the *X*-phase, each Ca site is surrounded by seven sulfur atoms forming a distorted enneahedron. Each of the involved sulfur atoms is shared by the adjacent one or two Ca sites and by two Ga sites. The Ca—S distances range from 2.8261 (10) to 3.0823 (13) Å. Each Ga site is tetrahedrally surrounded by sulfur atoms which are also shared by adjacent two Ca sites and two Ga sites. The Ga—S distances and the S—Ga—S angles range from 2.2254 (11) to 2.3116 (11) Å and from 93.54 (3) to 124.12 (3) °, respectively. In the orthorhombic phase, each of the three Ca sites and the two Ga sites is in an eightfold and fourfold coordination environment, respectively, by sulfur atoms. Each of the sulfur atoms of these sites is shared by adjacent two Ca sites and two Ga sites. The Ca—S distances in this polymorph range from 2.970 to 3.130 Å. The Ga—S distances and the S—Ga—S angles range from 2.240 to 2.318 Å and from 96.6 to 125°. It should be noted that the coordination number of the Ca sites in the *X*-phase is reduced by one compared to that of the orthorhombic phase, while the Ga sites are tetrahedrally surrounded in both phases. Nevertheless, the framework built up from the GaS_4_ tetrahedra and their connection modes (corner and edge-sharing) is quite different in the two phases.

The intensities of photoluminescence from Mn^2+^ and Ce^3+^ in the *X*-phase were about four and eight times higher than those in the orthorhombic phase. These emission peaks shifted toward the high energy side in comparison with those in the orthorhombic phase. Mn^2+^ and Ce^3+^ ions are known to be very sensitive with respect to the crystal field (Peters & Baglio, 1972; Provenzano & White, 1991; Barthou *et al.*, 1994; Wang *et al.*, 2003). Thus the observed shifts of the emission peaks are assumed to be caused by a weakened crystal field originating from the decrease in the coordination number of the Ca sites in the *X*-phase.

### Experimental

A mixture of CaS (4 N) and Ga_2_S_3_ (6 N) was used as a starting material added with a small amount of a rare earth element (REE) or a transition metal (TM) as an activator. It was weighed to 0.5 g in total according to the formula Ca_1-_*_x_M_x_*Ga_2_S_4_ (*M* = REE or TM) (Boitier *et al.*, 2009; Obonai *et al.*, 2009). The mixture was carefully stirred to be homogenized under Ar atmosphere, and then sealed in a silica glass capsule under vacuum of 10 ^-4^ Pa, where the inner surface of the capsule was coated with a carbon film to prevent the reaction between the starting materials and the container. It was sintered at 1411 K for 1 h. The *X*-phase samples were sometimes and unexpectedly synthesized and the detailed condition how and when the *X*-phase emerges has not been clarified yet.

### Refinement

Freeing of the site occupation factors for the metal and the sulfur atoms revealed no noticeable incorporation of the dopant metals or of vacancies at the S positions. The highest difference peak of 2.64 e Å^-3^ is located 1.40 Å from the Ga4 atom; the deepest hole (-3.11 e Å^-3^) is 1.39 Å from the Ga4 atom.

### Crystal data

**Table:**

CaGa_2_S_4_	*F*(000) = 1168.00
*M**_r_* = 307.76	*D*_x_ = 3.237 Mg m^−^^3^
Monoclinic, *P*2_1_/*a*	Mo *K*α radiation, λ = 0.71075 Å
Hall symbol: -P 2yab	Cell parameters from 3521 reflections
*a* = 6.841 (3) Å	θ = 3.2–27.5°
*b* = 27.069 (9) Å	µ = 10.52 mm^−^^1^
*c* = 7.231 (3) Å	*T* = 270 K
β = 109.413 (5)°	Block, colorless
*V* = 1262.9 (8) Å^3^	0.10 × 0.10 × 0.05 mm
*Z* = 8	

### Data collection

**Table:**

Rigaku Saturn724+ diffractometer	2869 independent reflections
Graphite monochromator	256 reflections with *F*^2^ > 2σ(*F*^2^)
Detector resolution: 7.31 pixels mm^-1^	*R*_int_ = 0.047
ω scans	θ_max_ = 27.5°
Absorption correction: multi-scan (*REQABS*; Jacobson, 1998)	*h* = −8→6
*T*_min_ = 0.417, *T*_max_ = 0.591	*k* = −35→26
8869 measured reflections	*l* = −9→9

### Refinement

**Table:**

Refinement on *F*^2^	Primary atom site location: structure-invariant direct methods
*R*[*F*^2^ > 2σ(*F*^2^)] = 0.038	Secondary atom site location: difference Fourier map
*wR*(*F*^2^) = 0.050	Chebychev polynomial *w*ith 3 parameters (Carruthers & Watkin, 1979) 6334.0000 8721.6900 0.0000
*S* = 1.21	(Δ/σ)_max_ < 0.001
2869 reflections	Δρ_max_ = 2.64 e Å^−^^3^
127 parameters	Δρ_min_ = −3.10 e Å^−^^3^

### Special details

**Table:**

Geometry. ENTER SPECIAL DETAILS OF THE MOLECULAR GEOMETRY
Refinement. Refinement was performed using reflections with *F*^2^ > 2.0 σ(*F*^2^). The weighted *R*-factor (*wR*) and goodness of fit (*S*) are based on *F*^2^. *R*-factor (gt) are based on *F*. The threshold expression of *F*^2^ > 2.0 σ(*F*^2^) is used only for calculating *R*-factor (gt).

### Fractional atomic coordinates and isotropic or equivalent isotropic displacement parameters (Å^2^)

**Table:**

	*x*	*y*	*z*	*U*_iso_*/*U*_eq_	
Ga1	0.41763 (6)	0.234320 (10)	0.54983 (6)	0.01027 (10)	
Ga2	0.63163 (6)	0.150170 (10)	0.29040 (6)	0.01047 (10)	
Ga3	−0.05236 (6)	0.052250 (10)	0.27118 (6)	0.01054 (10)	
Ga4	0.23350 (6)	0.064220 (10)	0.01510 (6)	0.01095 (10)	
Ca1	−0.44242 (12)	0.07086 (3)	−0.32662 (11)	0.0133 (2)	
Ca2	0.09658 (12)	0.19323 (3)	0.04330 (11)	0.0145 (2)	
S1	−0.08531 (14)	0.09720 (3)	−0.01062 (13)	0.0114 (2)	
S2	−0.13487 (14)	0.09906 (4)	0.49207 (13)	0.0125 (2)	
S3	0.24485 (13)	0.25319 (4)	0.75754 (13)	0.0128 (2)	
S4	0.37219 (14)	0.15701 (3)	0.42780 (13)	0.0115 (2)	
S5	0.28330 (14)	0.02921 (3)	0.31401 (13)	0.0112 (2)	
S6	0.30654 (14)	0.27904 (3)	0.26168 (13)	0.0114 (2)	
S7	−0.26440 (15)	−0.01372 (4)	0.21648 (14)	0.0157 (2)	
S8	0.42875 (14)	0.13001 (3)	−0.02150 (13)	0.0118 (2)	

### Atomic displacement parameters (Å^2^)

**Table:**

	*U*^11^	*U*^22^	*U*^33^	*U*^12^	*U*^13^	*U*^23^
Ga1	0.0099 (2)	0.0137 (2)	0.0084 (2)	0.00054 (16)	0.00453 (16)	−0.00112 (16)
Ga2	0.0109 (2)	0.0120 (2)	0.0094 (2)	0.00015 (16)	0.00450 (16)	−0.00010 (16)
Ga3	0.0111 (2)	0.0126 (2)	0.0100 (2)	0.00015 (16)	0.00645 (16)	−0.00067 (16)
Ga4	0.0114 (2)	0.0141 (2)	0.0091 (2)	−0.00123 (17)	0.00581 (16)	−0.00087 (16)
Ca1	0.0127 (4)	0.0164 (4)	0.0114 (3)	−0.0016 (3)	0.0049 (3)	0.0007 (3)
Ca2	0.0146 (4)	0.0145 (4)	0.0140 (4)	−0.0003 (3)	0.0044 (3)	−0.0005 (3)
S1	0.0106 (4)	0.0161 (4)	0.0078 (4)	0.0007 (3)	0.0037 (3)	0.0016 (3)
S2	0.0153 (4)	0.0151 (5)	0.0084 (4)	0.0037 (3)	0.0056 (3)	−0.0004 (3)
S3	0.0074 (4)	0.0234 (5)	0.0088 (4)	0.0017 (3)	0.0043 (3)	−0.0004 (3)
S4	0.0134 (4)	0.0118 (4)	0.0114 (4)	−0.0002 (3)	0.0069 (3)	−0.0008 (3)
S5	0.0118 (4)	0.0151 (4)	0.0070 (4)	0.0024 (3)	0.0035 (3)	0.0015 (3)
S6	0.0135 (5)	0.0130 (4)	0.0094 (4)	0.0015 (3)	0.0062 (3)	0.0006 (3)
S7	0.0197 (5)	0.0167 (5)	0.0168 (5)	−0.0072 (3)	0.0142 (4)	−0.0073 (3)
S8	0.0124 (4)	0.0145 (4)	0.0093 (4)	−0.0029 (3)	0.0049 (3)	−0.0019 (3)

### Geometric parameters (Å, º)

**Table:**

Ga1—S3	2.2569 (11)	Ga4—S8	2.2936 (9)
Ga1—S3^i^	2.2685 (8)	Ca1—S1	2.8261 (10)
Ga1—S4	2.2522 (8)	Ca1—S2^iv^	2.9260 (14)
Ga1—S6	2.3090 (9)	Ca1—S4^v^	2.9500 (11)
Ga2—S2^ii^	2.2458 (9)	Ca1—S5^v^	2.8814 (10)
Ga2—S4	2.3116 (11)	Ca1—S5^iii^	2.9095 (11)
Ga2—S6^i^	2.3048 (9)	Ca1—S7^vi^	2.8480 (14)
Ga2—S8	2.2868 (8)	Ca1—S8^vii^	3.0823 (13)
Ga3—S1	2.3190 (10)	Ca2—S1	2.8522 (11)
Ga3—S2	2.2526 (11)	Ca2—S3^iv^	3.0538 (13)
Ga3—S5	2.2989 (10)	Ca2—S3^viii^	2.9762 (10)
Ga3—S7	2.2513 (10)	Ca2—S4	2.9599 (10)
Ga4—S1	2.3062 (10)	Ca2—S6	2.9059 (11)
Ga4—S5	2.2795 (10)	Ca2—S6^ix^	3.0127 (14)
Ga4—S7^iii^	2.2254 (11)	Ca2—S8	3.0033 (13)
			
S5···S7^x^	3.4654 (14)	S7···S5^x^	3.4654 (14)
			
S3—Ga1—S3^i^	98.37 (3)	S3^iv^—Ca2—S6^ix^	130.31 (3)
S3—Ga1—S4	115.87 (4)	S3^iv^—Ca2—S8	76.03 (3)
S3—Ga1—S6	113.19 (3)	S3^viii^—Ca2—S4	158.30 (4)
S3^i^—Ga1—S4	112.04 (3)	S3^viii^—Ca2—S6	97.31 (3)
S3^i^—Ga1—S6	118.40 (3)	S3^viii^—Ca2—S6^ix^	73.81 (3)
S4—Ga1—S6	99.93 (3)	S3^viii^—Ca2—S8	130.42 (3)
S2^ii^—Ga2—S4	104.61 (3)	S4—Ca2—S6	73.08 (2)
S2^ii^—Ga2—S6^i^	106.99 (3)	S4—Ca2—S6^ix^	85.13 (3)
S2^ii^—Ga2—S8	124.12 (3)	S4—Ca2—S8	71.28 (3)
S4—Ga2—S6^i^	117.90 (3)	S6—Ca2—S6^ix^	78.97 (3)
S4—Ga2—S8	98.16 (3)	S6—Ca2—S8	106.10 (3)
S6^i^—Ga2—S8	105.82 (3)	S6^ix^—Ca2—S8	152.63 (3)
S1—Ga3—S2	110.72 (3)	Ga3—S1—Ga4	85.00 (3)
S1—Ga3—S5	93.54 (3)	Ga3—S1—Ca1	112.55 (3)
S1—Ga3—S7	113.14 (3)	Ga3—S1—Ca2	116.58 (3)
S2—Ga3—S5	122.11 (3)	Ga4—S1—Ca1	120.51 (4)
S2—Ga3—S7	105.79 (4)	Ga4—S1—Ca2	89.07 (3)
S5—Ga3—S7	111.40 (3)	Ca1—S1—Ca2	124.13 (3)
S1—Ga4—S5	94.40 (3)	Ga2^vii^—S2—Ga3	100.25 (3)
S1—Ga4—S7^iii^	120.06 (3)	Ga2^vii^—S2—Ca1^xi^	89.39 (3)
S1—Ga4—S8	105.16 (3)	Ga3—S2—Ca1^xi^	123.91 (4)
S5—Ga4—S7^iii^	115.87 (3)	Ga1—S3—Ga1^ix^	102.30 (3)
S5—Ga4—S8	121.53 (3)	Ga1—S3—Ca2^xi^	134.04 (4)
S7^iii^—Ga4—S8	100.69 (4)	Ga1—S3—Ca2^xii^	95.21 (3)
S1—Ca1—S2^iv^	74.89 (3)	Ga1^ix^—S3—Ca2^xi^	92.90 (3)
S1—Ca1—S4^v^	111.74 (3)	Ga1^ix^—S3—Ca2^xii^	141.19 (4)
S1—Ca1—S5^v^	163.07 (4)	Ca2^xi^—S3—Ca2^xii^	99.38 (3)
S1—Ca1—S5^iii^	89.75 (3)	Ga1—S4—Ga2	102.54 (3)
S1—Ca1—S7^vi^	114.26 (3)	Ga1—S4—Ca1^xiii^	121.53 (3)
S1—Ca1—S8^vii^	70.50 (3)	Ga1—S4—Ca2	91.27 (3)
S2^iv^—Ca1—S4^v^	75.72 (3)	Ga2—S4—Ca1^xiii^	87.56 (3)
S2^iv^—Ca1—S5^v^	92.74 (3)	Ga2—S4—Ca2	87.89 (3)
S2^iv^—Ca1—S5^iii^	86.54 (3)	Ca1^xiii^—S4—Ca2	147.08 (3)
S2^iv^—Ca1—S7^vi^	160.92 (4)	Ga3—S5—Ga4	86.08 (3)
S2^iv^—Ca1—S8^vii^	127.19 (3)	Ga3—S5—Ca1^xiii^	109.71 (4)
S4^v^—Ca1—S5^v^	75.27 (2)	Ga3—S5—Ca1^iii^	127.11 (3)
S4^v^—Ca1—S5^iii^	146.76 (4)	Ga4—S5—Ca1^xiii^	122.66 (3)
S4^v^—Ca1—S7^vi^	113.32 (3)	Ga4—S5—Ca1^iii^	110.69 (4)
S4^v^—Ca1—S8^vii^	81.50 (3)	Ca1^xiii^—S5—Ca1^iii^	102.20 (3)
S5^v^—Ca1—S5^iii^	77.80 (3)	Ga1—S6—Ga2^ix^	112.31 (4)
S5^v^—Ca1—S7^vi^	74.43 (3)	Ga1—S6—Ca2	91.50 (3)
S5^v^—Ca1—S8^vii^	126.39 (3)	Ga1—S6—Ca2^i^	121.80 (3)
S5^iii^—Ca1—S7^vi^	77.09 (3)	Ga2^ix^—S6—Ca2	122.40 (3)
S5^iii^—Ca1—S8^vii^	130.86 (3)	Ga2^ix^—S6—Ca2^i^	106.99 (3)
S7^vi^—Ca1—S8^vii^	71.73 (3)	Ca2—S6—Ca2^i^	101.95 (3)
S1—Ca2—S3^iv^	128.30 (3)	Ga3—S7—Ga4^iii^	113.74 (5)
S1—Ca2—S3^viii^	97.70 (3)	Ga3—S7—Ca1^vi^	148.67 (5)
S1—Ca2—S4	86.52 (3)	Ga4^iii^—S7—Ca1^vi^	97.39 (4)
S1—Ca2—S6	156.52 (4)	Ga2—S8—Ga4	104.91 (4)
S1—Ca2—S6^ix^	88.01 (3)	Ga2—S8—Ca1^ii^	127.28 (3)
S1—Ca2—S8	77.16 (3)	Ga2—S8—Ca2	87.30 (3)
S3^iv^—Ca2—S3^viii^	69.21 (3)	Ga4—S8—Ca1^ii^	89.73 (3)
S3^iv^—Ca2—S4	124.09 (3)	Ga4—S8—Ca2	85.67 (3)
S3^iv^—Ca2—S6	74.18 (3)	Ca1^ii^—S8—Ca2	144.97 (3)
			
S3—Ga1—S3^i^—Ga1^i^	−153.70 (4)	S5^v^—Ca1—S2^iv^—Ga3^iv^	−38.96 (4)
S3—Ga1—S3^i^—Ca2^xii^	−17.36 (4)	S2^iv^—Ca1—S5^iii^—Ga3^iii^	33.05 (5)
S3^i^—Ga1—S3—Ga1^ix^	163.03 (4)	S2^iv^—Ca1—S5^iii^—Ga4^iii^	134.24 (3)
S3^i^—Ga1—S3—Ca2^xi^	−90.54 (5)	S5^iii^—Ca1—S2^iv^—Ga3^iv^	38.63 (4)
S3^i^—Ga1—S3—Ca2^xii^	17.88 (4)	S2^iv^—Ca1—S7^vi^—Ga3^vi^	−17.88 (16)
S3—Ga1—S4—Ga2	−167.10 (3)	S7^vi^—Ca1—S2^iv^—Ga3^iv^	7.90 (13)
S3—Ga1—S4—Ca2	104.78 (3)	S2^iv^—Ca1—S8^vii^—Ga2^vii^	−64.03 (6)
S3—Ga1—S4—Ca1^xiii^	−72.20 (5)	S2^iv^—Ca1—S8^vii^—Ga4^vii^	−172.68 (3)
S4—Ga1—S3—Ga1^ix^	−77.44 (4)	S2^iv^—Ca1—S8^vii^—Ca2^vii^	105.26 (6)
S4—Ga1—S3—Ca2^xi^	28.99 (5)	S8^vii^—Ca1—S2^iv^—Ga3^iv^	179.61 (3)
S4—Ga1—S3—Ca2^xii^	137.40 (3)	S4^v^—Ca1—S5^v^—Ga3^v^	−62.52 (4)
S3—Ga1—S6—Ca2	−106.34 (3)	S4^v^—Ca1—S5^v^—Ga4^v^	35.72 (5)
S3—Ga1—S6—Ga2^ix^	19.83 (5)	S5^v^—Ca1—S4^v^—Ga1^v^	170.74 (5)
S3—Ga1—S6—Ca2^i^	148.59 (4)	S5^v^—Ca1—S4^v^—Ga2^v^	−86.03 (3)
S6—Ga1—S3—Ga1^ix^	37.16 (5)	S5^v^—Ca1—S4^v^—Ca2^v^	−3.70 (8)
S6—Ga1—S3—Ca2^xi^	143.58 (4)	S4^v^—Ca1—S5^iii^—Ga3^iii^	90.16 (7)
S6—Ga1—S3—Ca2^xii^	−108.00 (3)	S4^v^—Ca1—S5^iii^—Ga4^iii^	−168.64 (6)
S3^i^—Ga1—S4—Ga2	−55.34 (4)	S5^iii^—Ca1—S4^v^—Ga1^v^	−152.37 (6)
S3^i^—Ga1—S4—Ca2	−143.46 (3)	S5^iii^—Ca1—S4^v^—Ga2^v^	−49.14 (7)
S3^i^—Ga1—S4—Ca1^xiii^	39.56 (6)	S5^iii^—Ca1—S4^v^—Ca2^v^	33.19 (13)
S4—Ga1—S3^i^—Ga1^i^	83.93 (5)	S4^v^—Ca1—S7^vi^—Ga3^vi^	97.24 (8)
S4—Ga1—S3^i^—Ca2^xii^	−139.73 (3)	S7^vi^—Ca1—S4^v^—Ga1^v^	105.29 (5)
S3^i^—Ga1—S6—Ca2	139.35 (4)	S7^vi^—Ca1—S4^v^—Ga2^v^	−151.48 (3)
S3^i^—Ga1—S6—Ga2^ix^	−94.47 (5)	S7^vi^—Ca1—S4^v^—Ca2^v^	−69.15 (9)
S3^i^—Ga1—S6—Ca2^i^	34.28 (5)	S4^v^—Ca1—S8^vii^—Ga2^vii^	−128.79 (4)
S6—Ga1—S3^i^—Ga1^i^	−31.56 (5)	S4^v^—Ca1—S8^vii^—Ga4^vii^	122.55 (3)
S6—Ga1—S3^i^—Ca2^xii^	104.78 (4)	S4^v^—Ca1—S8^vii^—Ca2^vii^	40.50 (7)
S4—Ga1—S6—Ca2	17.50 (4)	S8^vii^—Ca1—S4^v^—Ga1^v^	39.47 (5)
S4—Ga1—S6—Ga2^ix^	143.67 (4)	S8^vii^—Ca1—S4^v^—Ga2^v^	142.70 (3)
S4—Ga1—S6—Ca2^i^	−87.57 (4)	S8^vii^—Ca1—S4^v^—Ca2^v^	−134.97 (8)
S6—Ga1—S4—Ga2	70.95 (3)	S5^v^—Ca1—S5^iii^—Ga3^iii^	126.60 (5)
S6—Ga1—S4—Ca2	−17.17 (4)	S5^v^—Ca1—S5^iii^—Ga4^iii^	−132.21 (4)
S6—Ga1—S4—Ca1^xiii^	165.85 (4)	S5^iii^—Ca1—S5^v^—Ga3^v^	137.15 (4)
S2^ii^—Ga2—S4—Ga1	107.72 (3)	S5^iii^—Ca1—S5^v^—Ga4^v^	−124.60 (5)
S2^ii^—Ga2—S4—Ca2	−161.45 (3)	S5^v^—Ca1—S7^vi^—Ga3^vi^	31.29 (8)
S2^ii^—Ga2—S4—Ca1^xiii^	−14.07 (3)	S7^vi^—Ca1—S5^v^—Ga3^v^	57.35 (4)
S4—Ga2—S2^ii^—Ga3^ii^	138.58 (3)	S7^vi^—Ca1—S5^v^—Ga4^v^	155.60 (5)
S2^ii^—Ga2—S6^i^—Ga1^i^	−68.55 (5)	S5^v^—Ca1—S8^vii^—Ga2^vii^	166.65 (4)
S2^ii^—Ga2—S6^i^—Ca2^i^	−175.65 (4)	S5^v^—Ca1—S8^vii^—Ga4^vii^	58.00 (4)
S6^i^—Ga2—S2^ii^—Ga3^ii^	−95.60 (4)	S5^v^—Ca1—S8^vii^—Ca2^vii^	−24.06 (9)
S2^ii^—Ga2—S8—Ga4	61.50 (6)	S8^vii^—Ca1—S5^v^—Ga3^v^	4.92 (6)
S2^ii^—Ga2—S8—Ca2	146.29 (4)	S8^vii^—Ca1—S5^v^—Ga4^v^	103.16 (5)
S2^ii^—Ga2—S8—Ca1^ii^	−39.84 (7)	S5^iii^—Ca1—S7^vi^—Ga3^vi^	−49.43 (8)
S8—Ga2—S2^ii^—Ga3^ii^	27.86 (6)	S7^vi^—Ca1—S5^iii^—Ga3^iii^	−156.82 (5)
S4—Ga2—S6^i^—Ga1^i^	48.85 (4)	S7^vi^—Ca1—S5^iii^—Ga4^iii^	−55.63 (3)
S4—Ga2—S6^i^—Ca2^i^	−58.25 (5)	S5^iii^—Ca1—S8^vii^—Ga2^vii^	59.76 (6)
S6^i^—Ga2—S4—Ga1	−10.94 (4)	S5^iii^—Ca1—S8^vii^—Ga4^vii^	−48.89 (4)
S6^i^—Ga2—S4—Ca2	79.88 (3)	S5^iii^—Ca1—S8^vii^—Ca2^vii^	−130.95 (6)
S6^i^—Ga2—S4—Ca1^xiii^	−132.73 (3)	S8^vii^—Ca1—S5^iii^—Ga3^iii^	−105.41 (6)
S4—Ga2—S8—Ga4	−52.39 (4)	S8^vii^—Ca1—S5^iii^—Ga4^iii^	−4.21 (6)
S4—Ga2—S8—Ca2	32.40 (3)	S7^vi^—Ca1—S8^vii^—Ga2^vii^	113.12 (4)
S4—Ga2—S8—Ca1^ii^	−153.73 (4)	S7^vi^—Ca1—S8^vii^—Ga4^vii^	4.47 (2)
S8—Ga2—S4—Ga1	−123.75 (3)	S7^vi^—Ca1—S8^vii^—Ca2^vii^	−77.59 (6)
S8—Ga2—S4—Ca2	−32.92 (3)	S8^vii^—Ca1—S7^vi^—Ga3^vi^	169.08 (8)
S8—Ga2—S4—Ca1^xiii^	114.47 (2)	S1—Ca2—S3^iv^—Ga1^iv^	−40.82 (6)
S6^i^—Ga2—S8—Ga4	−174.53 (3)	S3^iv^—Ca2—S1—Ga3	155.55 (4)
S6^i^—Ga2—S8—Ca2	−89.74 (3)	S3^iv^—Ca2—S1—Ga4	71.61 (4)
S6^i^—Ga2—S8—Ca1^ii^	84.13 (5)	S3^iv^—Ca2—S1—Ca1	−55.44 (7)
S8—Ga2—S6^i^—Ga1^i^	157.33 (3)	S1—Ca2—S3^viii^—Ga1^viii^	−114.19 (4)
S8—Ga2—S6^i^—Ca2^i^	50.23 (5)	S1—Ca2—S3^viii^—Ca2^ix^	109.55 (4)
S1—Ga3—S2—Ga2^vii^	30.00 (4)	S3^viii^—Ca2—S1—Ga3	−134.92 (4)
S1—Ga3—S2—Ca1^xi^	126.23 (4)	S3^viii^—Ca2—S1—Ga4	141.13 (3)
S2—Ga3—S1—Ga4	133.76 (3)	S3^viii^—Ca2—S1—Ca1	14.08 (6)
S2—Ga3—S1—Ca1	−105.20 (4)	S1—Ca2—S4—Ga1	−154.26 (4)
S2—Ga3—S1—Ca2	47.31 (5)	S1—Ca2—S4—Ga2	103.24 (3)
S1—Ga3—S5—Ga4	−7.49 (3)	S1—Ca2—S4—Ca1^xiii^	21.00 (9)
S1—Ga3—S5—Ca1^xiii^	115.89 (3)	S4—Ca2—S1—Ga3	23.68 (4)
S1—Ga3—S5—Ca1^iii^	−120.58 (4)	S4—Ca2—S1—Ga4	−60.26 (3)
S5—Ga3—S1—Ga4	7.41 (3)	S4—Ca2—S1—Ca1	172.69 (5)
S5—Ga3—S1—Ca1	128.44 (4)	S1—Ca2—S6—Ga1	17.08 (11)
S5—Ga3—S1—Ca2	−79.04 (4)	S1—Ca2—S6—Ga2^ix^	−100.72 (10)
S1—Ga3—S7—Ga4^iii^	54.29 (5)	S1—Ca2—S6—Ca2^i^	140.07 (9)
S1—Ga3—S7—Ca1^vi^	−132.51 (7)	S6—Ca2—S1—Ga3	−5.63 (13)
S7—Ga3—S1—Ga4	−107.68 (3)	S6—Ca2—S1—Ga4	−89.57 (10)
S7—Ga3—S1—Ca1	13.35 (5)	S6—Ca2—S1—Ca1	143.38 (8)
S7—Ga3—S1—Ca2	165.87 (4)	S1—Ca2—S6^ix^—Ga1^ix^	136.61 (3)
S2—Ga3—S5—Ga4	−124.70 (4)	S1—Ca2—S6^ix^—Ca2^ix^	−124.02 (3)
S2—Ga3—S5—Ca1^xiii^	−1.32 (5)	S6^ix^—Ca2—S1—Ga3	−61.56 (4)
S2—Ga3—S5—Ca1^iii^	122.21 (5)	S6^ix^—Ca2—S1—Ga4	−145.50 (3)
S5—Ga3—S2—Ga2^vii^	138.38 (3)	S6^ix^—Ca2—S1—Ca1	87.45 (5)
S5—Ga3—S2—Ca1^xi^	−125.39 (4)	S1—Ca2—S8—Ga2	−116.60 (3)
S2—Ga3—S7—Ga4^iii^	175.67 (3)	S1—Ca2—S8—Ga4	−11.41 (2)
S2—Ga3—S7—Ca1^vi^	−11.14 (9)	S1—Ca2—S8—Ca1^ii^	71.92 (6)
S7—Ga3—S2—Ga2^vii^	−92.93 (4)	S8—Ca2—S1—Ga3	95.26 (4)
S7—Ga3—S2—Ca1^xi^	3.30 (5)	S8—Ca2—S1—Ga4	11.32 (2)
S5—Ga3—S7—Ga4^iii^	−49.58 (5)	S8—Ca2—S1—Ca1	−115.73 (5)
S5—Ga3—S7—Ca1^vi^	123.62 (8)	S3^iv^—Ca2—S3^viii^—Ga1^viii^	13.97 (3)
S7—Ga3—S5—Ga4	109.08 (3)	S3^iv^—Ca2—S3^viii^—Ca2^ix^	−122.30 (3)
S7—Ga3—S5—Ca1^xiii^	−127.55 (4)	S3^viii^—Ca2—S3^iv^—Ga1^iv^	−124.07 (5)
S7—Ga3—S5—Ca1^iii^	−4.01 (6)	S3^iv^—Ca2—S4—Ga1	70.62 (5)
S1—Ga4—S5—Ga3	7.54 (3)	S3^iv^—Ca2—S4—Ga2	−31.88 (4)
S1—Ga4—S5—Ca1^xiii^	−103.42 (4)	S3^iv^—Ca2—S4—Ca1^xiii^	−114.12 (8)
S1—Ga4—S5—Ca1^iii^	135.89 (3)	S4—Ca2—S3^iv^—Ga1^iv^	75.36 (6)
S5—Ga4—S1—Ga3	−7.48 (3)	S3^iv^—Ca2—S6—Ga1	−147.66 (3)
S5—Ga4—S1—Ca1	−120.76 (4)	S3^iv^—Ca2—S6—Ga2^ix^	94.54 (4)
S5—Ga4—S1—Ca2	109.31 (3)	S3^iv^—Ca2—S6—Ca2^i^	−24.67 (3)
S1—Ga4—S7^iii^—Ga3^iii^	−55.77 (5)	S6—Ca2—S3^iv^—Ga1^iv^	131.51 (4)
S7^iii^—Ga4—S1—Ga3	115.90 (4)	S3^iv^—Ca2—S6^ix^—Ga1^ix^	−81.77 (5)
S7^iii^—Ga4—S1—Ca1	2.62 (6)	S3^iv^—Ca2—S6^ix^—Ca2^ix^	17.60 (5)
S7^iii^—Ga4—S1—Ca2	−127.31 (4)	S6^ix^—Ca2—S3^iv^—Ga1^iv^	−168.56 (4)
S1—Ga4—S8—Ga2	100.35 (4)	S3^iv^—Ca2—S8—Ga2	108.02 (3)
S1—Ga4—S8—Ca2	14.31 (3)	S3^iv^—Ca2—S8—Ga4	−146.80 (3)
S1—Ga4—S8—Ca1^ii^	−130.93 (3)	S3^iv^—Ca2—S8—Ca1^ii^	−63.47 (6)
S8—Ga4—S1—Ga3	−131.83 (3)	S8—Ca2—S3^iv^—Ga1^iv^	19.95 (4)
S8—Ga4—S1—Ca1	114.88 (4)	S3^viii^—Ca2—S4—Ga1	−52.14 (11)
S8—Ga4—S1—Ca2	−15.04 (3)	S3^viii^—Ca2—S4—Ga2	−154.64 (10)
S5—Ga4—S7^iii^—Ga3^iii^	56.51 (5)	S3^viii^—Ca2—S4—Ca1^xiii^	123.12 (10)
S7^iii^—Ga4—S5—Ga3	−119.03 (3)	S4—Ca2—S3^viii^—Ga1^viii^	145.80 (9)
S7^iii^—Ga4—S5—Ca1^xiii^	130.02 (4)	S4—Ca2—S3^viii^—Ca2^ix^	9.54 (12)
S7^iii^—Ga4—S5—Ca1^iii^	9.33 (5)	S3^viii^—Ca2—S6—Ga1	146.44 (3)
S5—Ga4—S8—Ga2	−4.69 (5)	S3^viii^—Ca2—S6—Ga2^ix^	28.64 (5)
S5—Ga4—S8—Ca2	−90.73 (4)	S3^viii^—Ca2—S6—Ca2^i^	−90.57 (3)
S5—Ga4—S8—Ca1^ii^	124.03 (3)	S6—Ca2—S3^viii^—Ga1^viii^	83.93 (4)
S8—Ga4—S5—Ga3	118.33 (3)	S6—Ca2—S3^viii^—Ca2^ix^	−52.34 (4)
S8—Ga4—S5—Ca1^xiii^	7.37 (6)	S3^viii^—Ca2—S6^ix^—Ga1^ix^	−124.78 (4)
S8—Ga4—S5—Ca1^iii^	−113.32 (4)	S3^viii^—Ca2—S6^ix^—Ca2^ix^	−25.41 (3)
S7^iii^—Ga4—S8—Ga2	−134.25 (3)	S6^ix^—Ca2—S3^viii^—Ga1^viii^	160.16 (3)
S7^iii^—Ga4—S8—Ca2	139.71 (3)	S6^ix^—Ca2—S3^viii^—Ca2^ix^	23.89 (3)
S7^iii^—Ga4—S8—Ca1^ii^	−5.53 (3)	S3^viii^—Ca2—S8—Ga2	154.19 (4)
S8—Ga4—S7^iii^—Ga3^iii^	−170.40 (3)	S3^viii^—Ca2—S8—Ga4	−100.62 (4)
S1—Ca1—S2^iv^—Ga3^iv^	129.31 (4)	S3^viii^—Ca2—S8—Ca1^ii^	−17.29 (9)
S2^iv^—Ca1—S1—Ga3	−148.00 (4)	S8—Ca2—S3^viii^—Ga1^viii^	−34.53 (5)
S2^iv^—Ca1—S1—Ga4	−50.23 (4)	S8—Ca2—S3^viii^—Ca2^ix^	−170.80 (4)
S2^iv^—Ca1—S1—Ca2	61.91 (5)	S4—Ca2—S6—Ga1	−13.63 (3)
S1—Ca1—S4^v^—Ga1^v^	−25.51 (7)	S4—Ca2—S6—Ga2^ix^	−131.43 (5)
S1—Ca1—S4^v^—Ga2^v^	77.72 (4)	S4—Ca2—S6—Ca2^i^	109.36 (3)
S1—Ca1—S4^v^—Ca2^v^	160.05 (7)	S6—Ca2—S4—Ga1	13.98 (3)
S4^v^—Ca1—S1—Ga3	144.50 (4)	S6—Ca2—S4—Ga2	−88.53 (3)
S4^v^—Ca1—S1—Ga4	−117.73 (4)	S6—Ca2—S4—Ca1^xiii^	−170.76 (8)
S4^v^—Ca1—S1—Ca2	−5.59 (7)	S4—Ca2—S6^ix^—Ga1^ix^	49.94 (4)
S1—Ca1—S5^v^—Ga3^v^	−179.34 (12)	S4—Ca2—S6^ix^—Ca2^ix^	149.31 (3)
S1—Ca1—S5^v^—Ga4^v^	−81.09 (14)	S6^ix^—Ca2—S4—Ga1	−65.96 (3)
S5^v^—Ca1—S1—Ga3	−103.81 (14)	S6^ix^—Ca2—S4—Ga2	−168.46 (3)
S5^v^—Ca1—S1—Ga4	−6.04 (16)	S6^ix^—Ca2—S4—Ca1^xiii^	109.30 (8)
S5^v^—Ca1—S1—Ca2	106.10 (13)	S4—Ca2—S8—Ga2	−25.94 (2)
S1—Ca1—S5^iii^—Ga3^iii^	−41.83 (5)	S4—Ca2—S8—Ga4	79.25 (3)
S1—Ca1—S5^iii^—Ga4^iii^	59.36 (4)	S4—Ca2—S8—Ca1^ii^	162.58 (6)
S5^iii^—Ca1—S1—Ga3	−61.51 (4)	S8—Ca2—S4—Ga1	128.13 (3)
S5^iii^—Ca1—S1—Ga4	36.26 (5)	S8—Ca2—S4—Ga2	25.63 (2)
S5^iii^—Ca1—S1—Ca2	148.40 (5)	S8—Ca2—S4—Ca1^xiii^	−56.61 (8)
S1—Ca1—S7^vi^—Ga3^vi^	−133.22 (7)	S6—Ca2—S6^ix^—Ga1^ix^	−23.74 (4)
S7^vi^—Ca1—S1—Ga3	14.18 (5)	S6—Ca2—S6^ix^—Ca2^ix^	75.63 (3)
S7^vi^—Ca1—S1—Ga4	111.95 (5)	S6^ix^—Ca2—S6—Ga1	74.59 (3)
S7^vi^—Ca1—S1—Ca2	−135.91 (5)	S6^ix^—Ca2—S6—Ga2^ix^	−43.21 (4)
S1—Ca1—S8^vii^—Ga2^vii^	−12.04 (4)	S6^ix^—Ca2—S6—Ca2^i^	−162.43 (3)
S1—Ca1—S8^vii^—Ga4^vii^	−120.69 (3)	S6—Ca2—S8—Ga2	39.37 (3)
S1—Ca1—S8^vii^—Ca2^vii^	157.25 (6)	S6—Ca2—S8—Ga4	144.56 (3)
S8^vii^—Ca1—S1—Ga3	72.56 (4)	S6—Ca2—S8—Ca1^ii^	−132.12 (6)
S8^vii^—Ca1—S1—Ga4	170.33 (4)	S8—Ca2—S6—Ga1	−77.71 (4)
S8^vii^—Ca1—S1—Ca2	−77.53 (5)	S8—Ca2—S6—Ga2^ix^	164.48 (4)
S2^iv^—Ca1—S4^v^—Ga1^v^	−92.49 (5)	S8—Ca2—S6—Ca2^i^	45.27 (3)
S2^iv^—Ca1—S4^v^—Ga2^v^	10.73 (2)	S6^ix^—Ca2—S8—Ga2	−57.78 (7)
S2^iv^—Ca1—S4^v^—Ca2^v^	93.07 (8)	S6^ix^—Ca2—S8—Ga4	47.41 (7)
S4^v^—Ca1—S2^iv^—Ga3^iv^	−113.01 (4)	S6^ix^—Ca2—S8—Ca1^ii^	130.74 (7)
S2^iv^—Ca1—S5^v^—Ga3^v^	−136.98 (3)	S8—Ca2—S6^ix^—Ga1^ix^	80.03 (8)
S2^iv^—Ca1—S5^v^—Ga4^v^	−38.74 (5)	S8—Ca2—S6^ix^—Ca2^ix^	179.40 (6)

Symmetry codes: (i) *x*+1/2, −*y*+1/2, *z*; (ii) *x*+1, *y*, *z*; (iii) −*x*, −*y*, −*z*; (iv) *x*, *y*, *z*−1; (v) *x*−1, *y*, *z*−1; (vi) −*x*−1, −*y*, −*z*; (vii) *x*−1, *y*, *z*; (viii) *x*−1/2, −*y*+1/2, *z*−1; (ix) *x*−1/2, −*y*+1/2, *z*; (x) −*x*, −*y*, −*z*+1; (xi) *x*, *y*, *z*+1; (xii) *x*+1/2, −*y*+1/2, *z*+1; (xiii) *x*+1, *y*, *z*+1.
